# Porto‐Sinusoidal Vascular Disorder‐Spectrum Portal Venopathy as a Structural Substrate for Portal Hypertension in Anti‐Centromere Antibody‐Positive Primary Biliary Cholangitis

**DOI:** 10.1111/pin.70179

**Published:** 2026-09-15

**Authors:** Yuya Ando, Suguru Mabuchi, Satoshi Nakayama, Arata Honda, Ryota Hokari, Kenichi Harada, Naoya Murashima

**Affiliations:** ^1^ Department of Gastroenterology National Defense Medical College Hospital Tokorozawa Saitama Japan; ^2^ Department of Gastroenterology Mishuku Hospital Meguro‐ku Tokyo Japan; ^3^ Department of General Medicine Institute of Science Tokyo Bunkyo‐ku Tokyo Japan; ^4^ Department of Human Pathology, Graduate School of Medical Sciences Kanazawa University Kanazawa Ishikawa Japan

**Keywords:** anti‐centromere antibody, hepatic venous pressure gradient, portal venopathy, porto‐sinusoidal vascular disorder, primary biliary cholangitis, spleen stiffness

## Abstract

Anti‐centromere antibody (ACA) positivity in primary biliary cholangitis (PBC) has been associated with a portal hypertension‐dominant clinical trajectory, even without advanced fibrosis. This study investigated whether porto‐sinusoidal vascular disorder (PSVD)‐spectrum portal venopathy may underlie this phenotype. This retrospective case‐series study included three ACA‐positive PBC‐spectrum patients who underwent liver biopsy. The clinical characteristics, imaging findings, liver and spleen stiffness, hepatic venography, and hepatic venous pressure gradient (HVPG) were evaluated. Liver specimens were assessed for PSVD‐spectrum portal venopathy and PBC‐related bile duct injury. All patients presented with PSVD‐spectrum portal venopathy on liver biopsy; PBC‐defining bile duct lesions were present in two patients, and the presence of ductular reaction was suggestive of a biliary disease but not diagnostic for PBC in one patient. All patients had manifestations of portal hypertension with preserved liver synthetic function and noncirrhotic liver stiffness. The portal sandwich sign was present in all patients. When available, spleen stiffness was markedly elevated, indicating spleen–liver stiffness dissociation. HVPG was heterogeneous, with normal values in two patients and marked elevation in one patient. In summary, PSVD‐spectrum portal venopathy may contribute to disproportionate portal hypertension in ACA‐positive PBC‐spectrum patients but constitutes a diagnostic gray zone because PBC is defined as exclusionary in PSVD definitions.

## Graphical abstract

Anti‐centromere antibody‐positive primary biliary cholangitis‐spectrum disease may present with portal hypertension disproportionate to liver stiffness. In this series of three patients, portal tract‐based histology showed portal venopathy with porto‐sinusoidal vascular disorder‐spectrum features, supported by venographic, elastographic, and hemodynamic findings. These three cases highlight a diagnostic gray zone between autoimmune cholangiopathy and porto‐sinusoidal vascular disorder‐like portal venous remodeling.

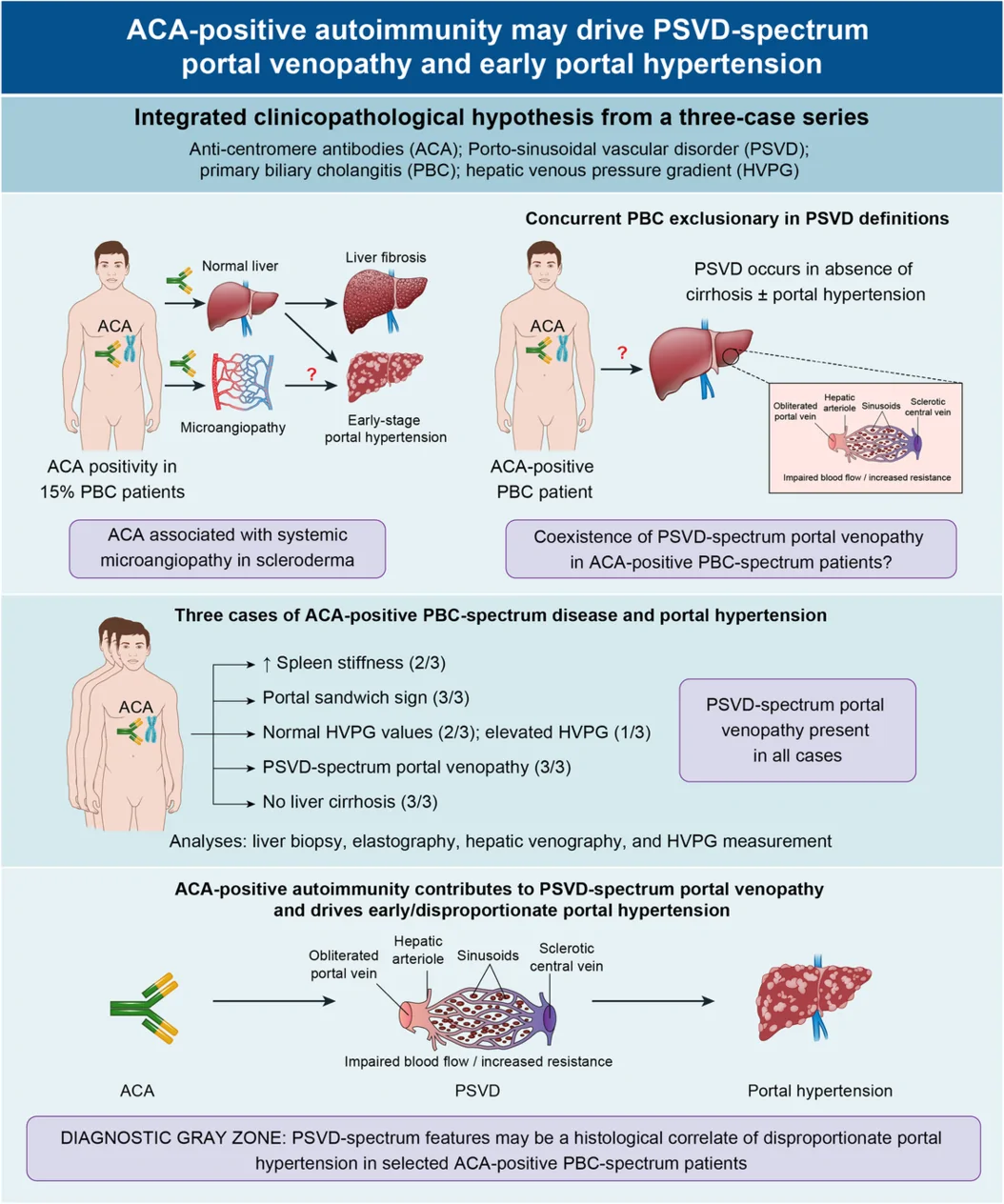

**Abbreviations**
2D‐SWE: two‐dimensional shear‐wave elastography
ACA: anti‐centromere antibody
ALP: alkaline phosphatase
AMA: anti‐mitochondrial antibody
AMA‐M2: anti‐mitochondrial antibody M2 subtype
ANA: anti‐nuclear antibody
CK7: cytokeratin 7
EV: esophageal varices
GGT: gamma‐glutamyl transferase
GV: gastric varices
HVPG: hepatic venous pressure gradient
IPH: idiopathic portal hypertension
lcSSc: limited cutaneous systemic sclerosis
LSM: liver stiffness measurement
MCTD: mixed connective tissue disease
NRH: nodular regenerative hyperplasia
PBC: primary biliary cholangitis
PH: portal hypertension
PHG: portal hypertensive gastropathy
PSVD: porto‐sinusoidal vascular disorder
SSc: systemic sclerosis
SSM: spleen stiffness measurement
VCTE: vibration‐controlled transient elastography
VV: veno‐venous

## 1 Introduction

Anti‐centromere antibody (ACA) positivity identifies a clinically relevant subset of primary biliary cholangitis (PBC), present in approximately 15% of patients with PBC [1], in which portal hypertension (PH) can become a dominant clinical problem at an early stage relative to fibrosis, distinct from a hepatic failure‐dominant course [2, 3]. We previously reported an ACA‐positive PBC patient with coexisting idiopathic portal hypertension (IPH) and noncirrhotic portal venopathy, raising the hypothesis that ACA‐associated systemic microangiopathy may contribute to portal venous remodeling beyond bile duct‐directed autoimmunity [4].

Porto‐sinusoidal vascular disorder (PSVD) is a clinicopathological entity characterized by lesions involving portal venules and/or sinusoids in the absence of cirrhosis, with or without manifestations of PH [5, 6, 7]. PSVD encompasses conditions historically labeled as IPH or noncirrhotic portal fibrosis and related entities, and is associated with immune‐mediated disorders, medications, hematological diseases, and prothrombotic states [6, 8]. However, because chronic cholestatic liver diseases are considered exclusionary conditions in formal PSVD diagnostic frameworks, no studies have systematically examined the coexistence of PSVD‐spectrum portal venopathy in ACA‐positive PBC‐spectrum patients.

Here, we report three cases in which PSVD‐spectrum portal venopathy may represent a structural substrate for disproportionate/early PH in ACA‐positive PBC‐spectrum disease. We describe three illustrative ACA‐positive patients with PBC‐spectrum disease and PH who underwent liver biopsy, elastography, hepatic venography, and HVPG measurement. Additionally, this report was not designed to estimate the prevalence of portal venopathy among ACA‐positive PBC patients or to establish a causal relationship between ACA positivity and portal venous remodeling.

## 2 Methods

We conducted a retrospective multicenter case‐series study from August 2021 to July 2023 across two institutions: Mishuku Hospital (including a previously published case [4]) and National Defense Medical College Hospital. Patients were eligible if they met the following criteria: (i) ACA positivity; (ii) definite or suspected PBC based on cholestatic liver biochemistry with anti‐mitochondrial antibody (AMA)/AMA‐M2 positivity and/or histology compatible with PBC; (iii) clinically significant PH (esophagogastric varices and splenomegaly); (iv) no radiological or histological evidence of cirrhosis; and (v) availability of PH work‐up, including elastography, hepatic venous pressure gradient (HVPG) measurement with hepatic venography, and liver biopsy. We also reviewed the absence of portal/splenic vein thrombosis, Budd–Chiari syndrome, congenital hepatic fibrosis, viral hepatitis, significant alcohol use, MASLD with advanced fibrosis, schistosomiasis, where epidemiologically relevant, hematologic disease, prothrombotic disorders, and exposure to drugs associated with PSVD.

We extracted the demographic characteristics, autoimmune comorbidities, laboratory test results, endoscopic findings, and imaging features, including the portal sandwich sign. Liver stiffness and spleen stiffness values were obtained using shear‐wave elastography or vibration‐controlled transient elastography, depending on institutional availability. HVPG was calculated as wedged hepatic venous pressure minus free hepatic venous pressure; repeated measurements were performed when feasible, and hepatic venography was evaluated for PSVD‐spectrum morphological patterns [5, 9]. Liver biopsy slides were centrally reviewed by a liver pathologist (K.H.), with a portal‐focused approach for PSVD‐spectrum portal venopathy (portal vein stenosis/obliteration, dilated/herniated portal vein, portal vein muscularization, portal fibrosis, nodular regenerative hyperplasia [NRH]) and PBC‐related bile duct injury/loss [5, 9].

Because chronic cholestatic liver diseases are listed as exclusion criteria in formal PSVD frameworks [5, 6], we did not aim to assign a definitive PSVD diagnosis. Instead, we used the descriptive term “PSVD‐spectrum portal venopathy” when biopsies demonstrated one or more of the following histological features [5]: (i) obliterative portal venopathy/portal vein branch narrowing or obliteration affecting ≥ 50% of evaluable portal tracts; (ii) abnormal portal tract vessels including muscularized portal venules or paraportal shunting vessels; (iii) features suggestive of NRH on reticulin staining; (iv) incomplete septal fibrosis. The study used de‐identified data and was conducted in accordance with institutional requirements. Written informed consent for publication was obtained as required.

## 3 Results

### 3.1 Overview

**Figure 1:**
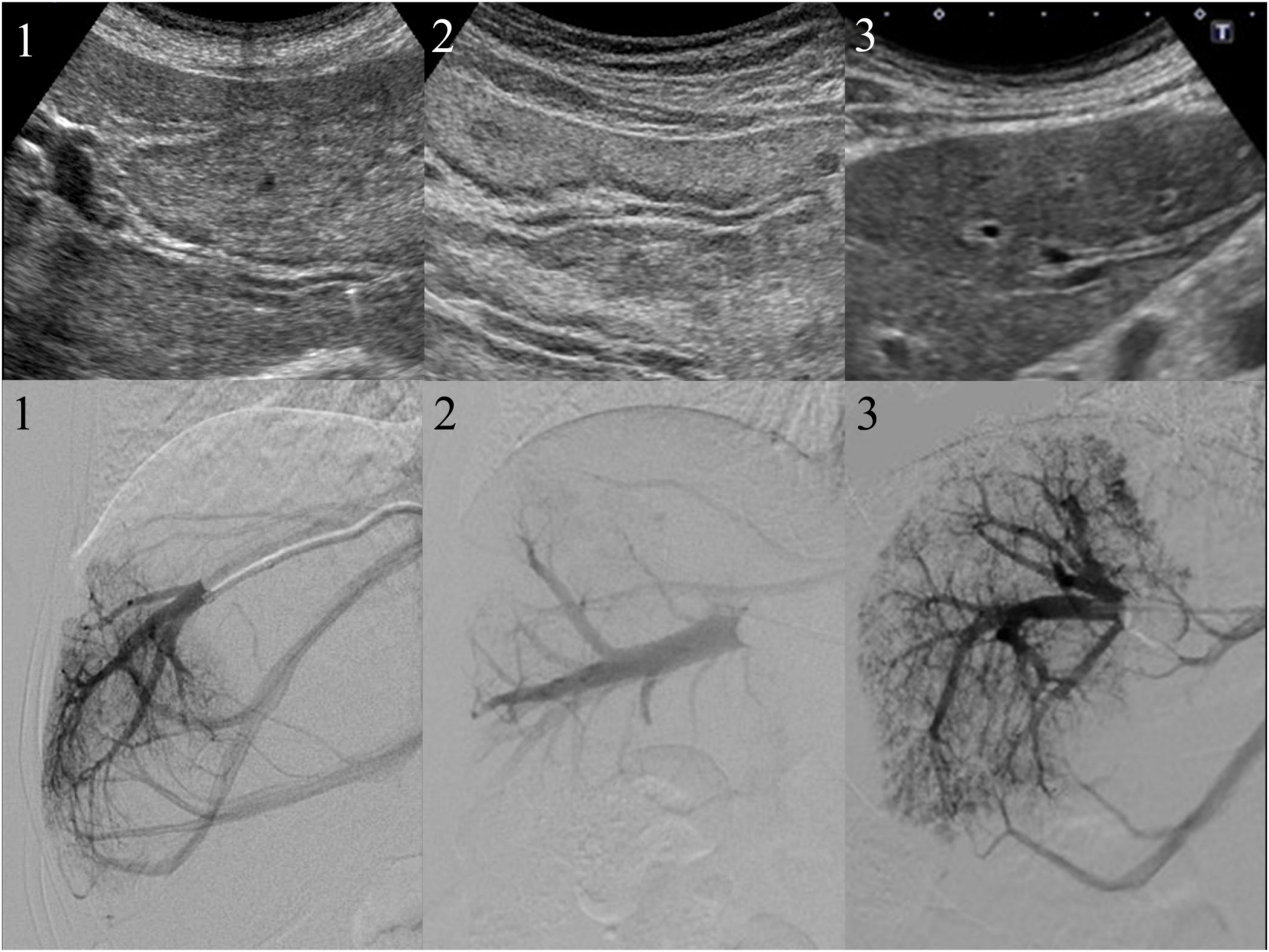
Upper: liver ultrasonography. The examinations revealed the “portal sandwich sign”. Lower: Hepatic venography. Hepatic venography shows anastomosis between hepatic veins. Case 1 is reproduced from Ando Y et al. Journal of Medical Case Reports. 2025;19:392, under CC BY‐NC‐ND 4.0.

Three ACA‐positive patients (two men, one woman; age range, 56–75 years) with PBC‐spectrum disease presented with clinically significant PH. The patient characteristics are summarized in Table 1, and additional detailed clinical, biochemical, serological, and hemodynamic findings are provided in the Supplementary Table. All patients had esophagogastric varices and splenomegaly with thrombocytopenia, while liver synthetic function was preserved. Liver stiffness measurement values were within a noncirrhotic range (8.5–9.3 kPa). The portal sandwich sign was identified on ultrasonography in all patients (Figure 1). Where available (two patients), spleen stiffness measurement values were markedly elevated (> 75 kPa [device upper limit: 75 kPa] and 108.2 kPa), demonstrating spleen–liver stiffness dissociation. Case vignettes are presented in Table 1.

**Table 1:** Clinical, biochemical, serological, and hemodynamic findings.

Variable	Case 1	Case 2	Case 3
Age/sex	57/F	75/M	56/M
Systemic autoimmune phenotype	None at presentation; subsequent lcSSc	lcSSc	MCTD‐spectrum autoimmunity with Sjögren syndrome
UDCA at assessment	Yes	Yes	No
AST, U/L	175	16	33
ALT, U/L	192	11	28
ALP, U/L	1019	98	103
GGT, U/L	396	16	73
Total bilirubin, mg/dL	1.26	1.5	0.45
Albumin, g/dL	4.2	3.8	4.1
Platelet count, ×10^9^/L	130	68	77
PT‐INR	0.96	1.19	0.92
IgM, mg/dL	613	203	189
ANA titer, pattern	1:1280, centromere	1:640, centromere	1:5120, speckled; cytoplasmic 1:640
ACA, IIF titer or IU/mL*	1:1280	258	> 500
AMA, IIF titer	1:160	< 1:20	< 1:20
AMA‐M2, index (cutoff < 7.0)	59.9	45.5	30.2
Key additional autoantibodies	—	—	Anti‐U1 RNP, > 550 IU/mL; anti‐SSA/Ro, > 1200 IU/mL
Varices	Present	Present	Present
Splenomegaly	Present	Present	Present
Portal sandwich sign	Present	Present	Present
LSM, kPa	8.5	9.3	8.5–9.0
SSM, kPa	NA	108.2	> 75
HVPG, mmHg	< 5	17 before and 13 after carvedilol	4–6
Hepatic venography	Weeping willow	Distal hepatic venous pruning	Veno‐venous shunts with peripheral pruning‐like changes

Abbreviations: ACA, anti‐centromere antibody; ALP, alkaline phosphatase; ALT, alanine aminotransferase; AMA, anti‐mitochondrial antibody; AMA‐M2, anti‐mitochondrial antibody M2 subtype; ANA, antinuclear antibody; AST, aspartate aminotransferase; GGT, gamma‐glutamyl transferase; HVPG, hepatic venous pressure gradient; IIF, indirect immunofluorescence; lcSSc, limited cutaneous systemic sclerosis; LSM, liver stiffness measurement; MCTD, mixed connective tissue disease; NA, not available; PT‐INR, prothrombin time–international normalized ratio; SSA/Ro, Sjögren syndrome‐related antigen A/Ro; SSM, spleen stiffness measurement; U1 RNP, U1 ribonucleoprotein; UDCA, ursodeoxycholic acid.

* For ACA measurements, indirect immunofluorescence was used in Case 1, with the result reported as a titer, whereas quantitative assays were used in Cases 2 and 3, with the results presented as IU/mL; therefore, the results are not directly comparable across the three cases.

### 3.2 Hemodynamics and Venography

HVPG values were heterogeneous. Two patients had normal HVPG values (< 5–6 mmHg), consistent with predominant presinusoidal PH, while the remaining patient had markedly elevated HVPG (17 mmHg) with a reduction to 13 mmHg after carvedilol initiation. Hepatic venography showed PSVD‐spectrum patterns: a “weeping willow” morphology in Case 1, distal hepatic venous pruning in Case 2, and veno‐venous shunts with peripheral pruning‐like changes in Case 3. The venographies are shown in Figure 1.

### 3.3 Histopathology

**Figure 2:**
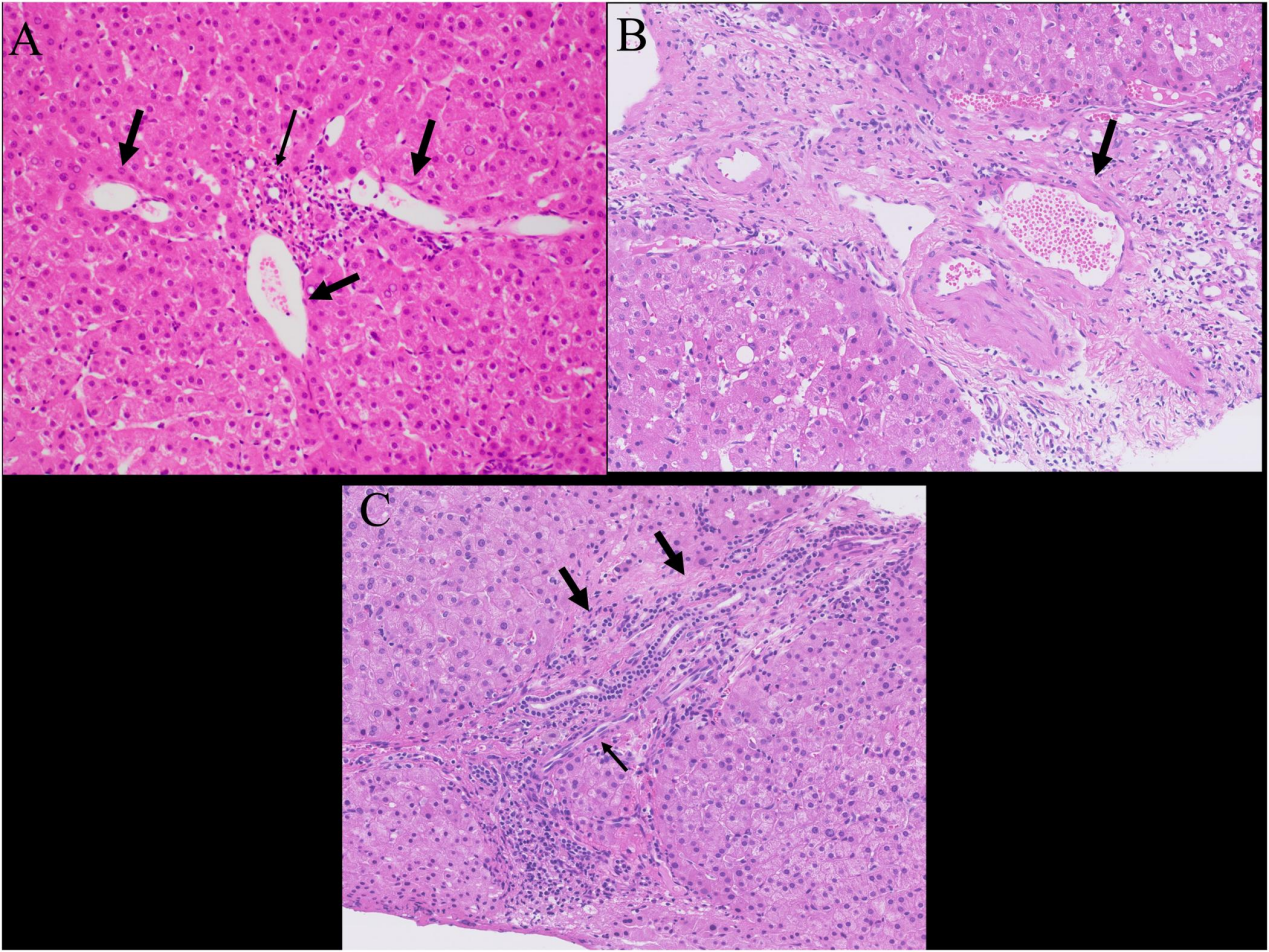
Hematoxylin‐and‐eosin staining of liver biopsy specimens at high magnification. (A) Case 1. In the small portal tract, interlobular bile ducts were preserved (thin arrow), while abnormal vessels (broad arrows) were found around the portal tract. Reproduced from Ando Y et al., Journal of Medical Case Reports, 2025;19:392, under CC BY‐NC‐ND 4.0. (B) Case 2. Portal vein muscularization (broad arrow) was found. (C) Case 3. Portal vein narrowing (thin arrow) and ductular reaction (broad arrows) were observed.

All patients demonstrated PSVD‐spectrum portal venopathy on liver biopsy examination. PBC‐defining bile duct lesions, such as cholangitis and bile duct loss, were confirmed in Cases 1 and 2, whereas Case 3 showed the presence of ductular reaction, suggestive of a type of biliary disease but not diagnostic for PBC. The three liver biopsy specimens are shown in Figure 2.

### 3.4 Case Vignettes

#### 3.4.1 Case 1 (Mishuku Hospital; Previously Published Case [4])

A 57‐year‐old Japanese woman with cholestasis and no prior diagnosis of autoimmune diseases was referred for evaluation. Laboratory tests showed marked cholestatic enzyme elevation (alkaline phosphatase, 1019 IU/L; γ‐GTP, 396 IU/L), mild thrombocytopenia (platelets, 13.0 × 10^4^/µL), and positivity for anti‐nuclear antibody (ANA) (1:1280), ACA (1:1280), and AMA (1:160). Abdominal ultrasonography demonstrated the portal sandwich sign. Two‐dimensional shear‐wave elastography (2D‐SWE) showed liver stiffness of 8.5 kPa (median). Endoscopy revealed esophageal varices, and computed tomography showed splenomegaly with esophageal and gastric varices. Hepatic venography demonstrated a “weeping willow” morphology. HVPG was < 5 mmHg (reference value). A 16‐mm liver biopsy revealed medium‐sized portal tracts disproportionate to the peripheral tissue, indicating progressive peripheral liver atrophy, but not cirrhosis. There were no clear histological findings of chronic nonsuppurative destructive cholangitis, but some septal bile ducts exhibited chronic cholangitis, consistent with early‐stage PBC, classified as CA2, HA 1, Stage 1 (fibrosis score 0, bile duct loss score 0, orcein‐positive granule score 0) [10]. In addition to the presence of characteristic abnormal vessels, portal vein stenosis was observed in four of five portal tracts, fulfilling the histological criteria for PSVD and indicating PSVD‐spectrum portal venopathy, although biliary diseases, including PBC, are considered exclusion criteria [7] (Figure 2A).

#### 3.4.2 Case 2 (Mishuku Hospital)

**Figure 3:**
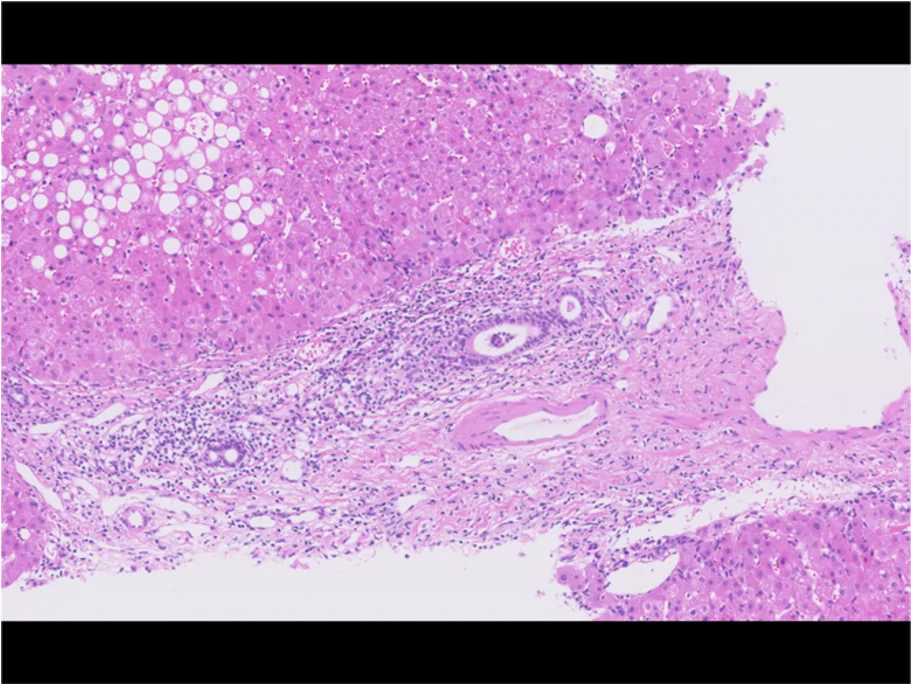
Hematoxylin‐and‐eosin staining of the liver biopsy specimen in Case 2. Chronic nonsuppurative destructive cholangitis compatible with PBC were observed.

A 75‐year‐old man with a remote diagnosis of limited cutaneous systemic sclerosis (lcSSc) and long‐standing AMA positivity was referred for prophylactic treatment of esophageal varices. Positivity was noted for ACA (258 IU/mL), ANA 1:640 (centromere pattern), and AMA‐M2 (45.5 index). Laboratory tests showed preserved liver function with mild thrombocytopenia (platelets 6.8 × 10^4^/µL); the serum AST, ALT, ALP, and gamma‐glutamyl transferase (GGT) levels were 16, 11, 98, and 16 U/L, respectively. Ultrasound showed the portal sandwich sign and splenomegaly. 2D‐SWE revealed liver stiffness of 9.3 kPa and spleen stiffness of 108.2 kPa. HVPG was 17 mmHg (median of five runs), with distal hepatic venous pruning on venography, but decreased to 13 mmHg after carvedilol initiation. Histologically, the lobular architecture was preserved, and no bridging fibrosis or cirrhosis was identified in the 7.5‐mm biopsy specimen. Although the specimen was shorter than the minimum total biopsy length recommended for the reliable exclusion of cirrhosis under the 2026 consensus framework [7], four portal tracts were identified, which is sufficient to rule out the presence of cirrhosis. Moreover, the radiological findings and low liver stiffness measurement did not suggest cirrhosis. The most striking portal tract finding was a muscularized portal venule (Figure 2B), which constitutes a major histological criterion for PSVD/NCPF [7]. Neither portal venule stenosis nor NRH was identified, although regenerative changes were present. Hypervascularized portal tracts characterized by increased small‐caliber vessels in portal tracts were also observed, although these findings are not included among the current major or minor histological criteria. In addition, chronic cholangitis and focal bile duct loss consistent with PBC were present (CA1, HA0, Stage 2 [fibrosis score 1, bile duct loss score 1, orcein‐positive granule score 0]) (Figure 3). Overall, the findings supported PBC with coexisting portal venopathy in the PSVD spectrum.

#### 3.4.3 Case 3 (National Defense Medical College Hospital)

A 56‐year‐old man with mixed connective tissue disease‐spectrum autoimmunity (anti‐RNP, ACA) and Sjögren syndrome (anti‐SSA) was referred to us because AMA‐M2 positivity (30.2 index) and persistently elevated ALP and γ‐GTP levels with unknown cause had raised suspicion for the presence of PBC. Endoscopy demonstrated esophageal varices (F2) with red signs, as well as portal hypertensive gastropathy and gastric varices. The serum AST, ALT, ALP, and GGT levels were 33, 28, 103, and 73 U/L, respectively. 2D‐SWE showed liver stiffness of 8.5–9.0 kPa and spleen stiffness > 75 kPa (device upper limit). Ultrasound demonstrated the portal sandwich sign. Hepatic venography showed veno‐venous shunts and peripheral pruning‐like changes; repeated HVPG measurements were 4–6 mmHg. A percutaneous biopsy procedure (18G; two cores) was performed, and the obtained liver specimens were consistent with two needle specimens with lengths of 13 and 7 mm. The lobular architecture was well preserved without bridging fibrosis, and no cirrhosis was noted. Portal tracts were mildly inflamed, with prominent ductular reaction. Portal vein branch narrowing was identified in three of five evaluable portal tracts (> 50%) (Figure 2C), fulfilling the histological criteria for PSVD‐spectrum portal venopathy [7]. Focal necrosis and sinusoidal fibrosis were noted, with reticulin staining suggesting an ill‐defined nodular tendency insufficient for the diagnosis of NRH. No florid duct lesions, bile duct loss, or ductopenia were identified. CK7 and orcein staining did not demonstrate features of chronic cholestasis but showed marked ductular reaction, indicating a biliary disease, including PBC (CA0, HA0, Stage 1) (Figure 2C). Overall, the pathological diagnosis was nonspecific reactive changes. However, from a clinical perspective, the patient was diagnosed with early‐stage PBC based on (i) the presence of AMA and (ii) elevated levels of bile duct enzymes (ALP, γ‐GTP) and total cholesterol (Supplementary Table), with slightly elevated IgM [11]. In summary, the findings supported PSVD with early PBC in the appropriate clinical context.

## 4 Discussion

In this multicenter series involving three ACA‐positive PBC‐spectrum patients, we consistently identified PSVD‐spectrum portal venopathy on portal‐focused liver histology, despite clinically significant PH and noncirrhotic liver stiffness. A convergent phenotype emerged: esophagogastric varices and splenomegaly with preserved synthetic function, reproducible portal sandwich sign, PSVD‐spectrum venographic abnormalities, and histologic portal vein branch narrowing/obliteration, with related portal tract vascular remodeling. Collectively, these observations raise the possibility that portal venopathy with PSVD‐spectrum features may serve as one histological correlate of disproportionate PH in selected ACA‐positive PBC‐spectrum patients [5, 6, 8].

Our findings closely align with the established clinicopathological profile of PSVD: a noncirrhotic PH disorder characterized by portal venular and/or sinusoidal lesions and a spectrum of portal‐flow disturbances [5]. PSVD includes symptoms previously classified as IPH and similar disorders, and is linked to immune‐mediated diseases, pharmacological agents, hematological disorders, and prothrombotic situations [6]. Key pathological lesions include obliterative portal venopathy/portal vein stenosis, abnormal portal tract vessels, and porto‐sinusoidal remodeling [6]. In our cases, portal‐focused histology demonstrated portal venopathy within this spectrum and imaging/hemodynamic patterns supporting a PSVD/IPH‐like phenotype. Although PSVD is classically considered a presinusoidal disorder with relatively low HVPG, a substantial subset of patients exhibit HVPG > 10 mmHg, particularly in ascites and more advanced disease, suggesting sinusoidal involvement in some PSVD phenotypes [8].

Our focus on the ACA is clinically motivated. While ACA are prevalent in lcSSc, they are associated with a PH‐dominant clinical trajectory in PBC, contrasting with anti‐gp210 positivity as a marker of hepatic failure‐type progression [2, 12]. Thus, ACA‐positive PBC may follow a PH‐dominant course that is not fully explained by parenchymal fibrosis alone. Mechanistically, ACA induce microvascular alterations and tissue remodeling, ultimately leading to fibrosis [13, 14]; therefore, ACA‐associated systemic autoimmunity provides a biologically plausible background for portal venous remodeling, although causality cannot be inferred from the present cases. Anti‐SSc autoantibodies, including ACA, can directly induce profibrotic activation in human dermal fibroblasts, while sera from SSc patients containing ACA can induce apoptosis‐related pathways in human dermal endothelial cells, consistent with antibody‐associated endothelial injury and impaired repair [13]. In parallel, IPH (PSVD‐spectrum archetypal entity) has long been linked with autoimmune comorbidities and immune dysregulation, such as skewing of the T‐cell receptor Vβ repertoire [15]. Pathological parallels between scleroderma and IPH, particularly shared small‐vessel injury and fibrogenic remodeling in systemic tissues and small portal tracts, also exist [16].

Viewed collectively, these cases may be better understood within a spectrum of autoantibody‐associated systemic microangiopathy rather than within a PBC‐specific framework. Case 3 is particularly informative because PSVD‐spectrum portal venopathy was evident despite the absence of morphological findings histologically characteristic of early or prefibrotic PBC. This observation raises two nonmutually exclusive possibilities: (i) portal venopathy may precede recognizable PBC‐related bile duct lesions in an AMA‐M2‐positive patient, or (ii) portal venopathy may arise independently as a hepatic manifestation of systemic immune‐mediated microvascular injury. In either scenario, portal venopathy may not merely represent a secondary consequence of established PBC‐related inflammation or fibrosis.

Moreover, all three patients had systemic sclerosis‐spectrum autoimmunity, and previous reports have linked ACA‐ or anti‐U1 RNP‐positive autoimmune disease to microvascular injury and IPH [13, 17, 18, 19, 20]. Portal venopathy may therefore represent a hepatic manifestation of systemic immune‐mediated microvascular injury, with or without PBC. In particular, ACA is a shared serological marker across PBC and lcSSc/CREST, and clonal T‐cell expansions have been reported in PBC–CREST overlap [21], whereas the association of ACA with pulmonary vascular disease in SSc supports a broader systemic vascular phenotype [22]. These observations support a strong rationale for classifying ACA‐mediated diseases under a unified category, with ACA as the central element. Although causality cannot be inferred from our series, this interpretation provides biological plausibility for a model in which ACA‐positive autoimmunity contributes to a PSVD‐spectrum portal venopathy phenotype driving early or disproportionate PH.

However, because PSVD diagnostic frameworks often treat chronic cholestatic liver diseases as exclusionary conditions [5, 6, 7], it is difficult to label patients as “PSVD + PBC,” even when portal venopathy is demonstrable. In PBC patients, an additional presinusoidal component of PH may not be assessed by HVPG, and thus HVPG may underestimate PH prevalence and severity [23]; notably, ACA status was not specifically addressed in the previous cohort. The Baveno VII consensus specifically cautions that HVPG may not capture an additional presinusoidal component in PBC, leading to underestimation of PH prevalence and severity; moreover, in chronic liver disease patients with clinical signs of PH but HVPG < 10 mmHg, PSVD should be actively ruled out [24]. This distinction is clinically relevant because PSVD can cause substantial PH‐related morbidity and impact long‐term outcomes, underscoring the importance of early recognition of PSVD‐spectrum features [8].

This report has limitations. The sample size was small and retrospective, and the PH workup was not uniform (spleen stiffness was unavailable in one case, and elastography platforms differed by center). Next, although Case 3 was diagnosed with PBC based on the clinical context [11], there was insufficient evidence to establish a histological diagnosis of PBC. Accordingly, this case should not be regarded as histologically established PBC. Nevertheless, the inclusion of this case is informative because it raises the possibility that PSVD‐spectrum portal venopathy may precede recognizable PBC morphology or occur independently of PBC in the setting of ACA‐positive systemic autoimmunity. In addition, because this was not a comparative study, we consider it a topic for future investigation to determine whether these alterations in vascular structure are commonly seen in ACA‐PBC and whether analogous vascular changes are absent in conventional ACA‐negative PBC. Finally, long‐term outcomes were not systematically assessed. Large multicenter cohorts with standardized hemodynamic and pathological reviews and longitudinal follow‐up are needed to clarify the prevalence, prognosis, and mechanisms of PSVD‐spectrum portal venopathy in ACA‐positive PBC‐spectrum disease.

In this clinicopathological report involving three ACA‐positive PBC‐spectrum patients, PSVD‐spectrum portal venopathy was consistently identified on portal‐focused histology, despite noncirrhotic liver stiffness and clinically significant PH. These findings suggest that PSVD‐spectrum portal venopathy may provide a structural substrate for early/disproportionate PH in a subset of ACA‐positive PBC, constituting a diagnostic gray zone because chronic cholestatic diseases are treated as exclusionary in current PSVD frameworks.

## Author Contributions

Yuya Ando, Suguru Mabuchi, Satoshi Nakayama, Arata Honda, Ryota Hokari, and Naoya Murashima were responsible for the clinical data collection and patient management. Kenichi Harada performed the central pathology review. Yuya Ando drafted the manuscript. All authors critically reviewed and approved the final version of the manuscript.

## Funding

The authors have nothing to report.

## Ethics Statement

Under the respective institutional policies of Mishuku Hospital and National Defense Medical College Hospital, formal ethical approval was not required for this article.

## Consent

Written informed consent for publication of clinical details and images was obtained from all patients.

## Conflicts of Interest

The authors declare no conflicts of interest.

## Supporting information


Supporting File

## Data Availability

The data that support the findings of this study are available on request from the corresponding author. The data are not publicly available due to privacy or ethical restrictions. The datasets generated and/or analyzed during the current study are not publicly available owing to patient privacy but are available from the corresponding author on reasonable request.
