# Supplementary material for: Porto‐Sinusoidal Vascular Disorder‐Spectrum Portal Venopathy as a Structural Substrate for Portal Hypertension in Anti‐Centromere Antibody‐Positive Primary Biliary Cholangitis
Source: Pathol Int. 2026 Sep 15;76(9):e70179. doi: 10.1111/pin.70179 (PMC13575599; doi:10.1111/pin.70179)
Supplement: Supplementary file 1 [file PIN-76-0-s001.docx]

# **Supplementary Table**

| **Table.** Detailed clinical, biochemical, and hemodynamic findings | | | | | | |
| --- | --- | --- | --- | --- | --- | --- |
| Value |  | Case 1 | Case 2 | Case 3 | Reference range | |
| Age/sex |  | 57/F | 75/M | 56/M |  |  |
| Biolchemical |  |  |  |  | Lower limit | Upper limit |
| Sodium (mEq/L) |  | 141 | 144 | 138 | 136 | 145 |
| Potassium (mEq/L) |  | 4.4 | 3.6 | 4.4 | 3.4 | 4.5 |
| Chloride (mEq/L) |  | 104 | 111 | 102 | 100 | 108 |
| Bloog Sugar (mg/dL) |  | 144 | 96 | 101 | 70 | 109 |
| Blood Urea Nitrogen (mg/dL) |  | 21 | 14 | 13 | 8 | 20 |
| Creatinine (mg/dL) |  | 0.68 | 1.4 | 1.03 | 0.4 | 0.8 |
| AST (IU/L) |  | 175 | 16 | 33 | 7 | 38 |
| ALT (IU/L) |  | 192 | 11 | 28 | 4 | 44 |
| Total bilirubin (mg/dL) |  | 1.26 | 1.5 | 0.45 | 0.3 | 1.2 |
| Albumin (g/dL) |  | 4.2 | 3.8 | 4.1 | 4.2 | 5.1 |
| LDH (IU/L) |  | 178 | 130 | 229 | 106 | 220 |
| ALP (IU/L) |  | 1,019 | 98 | 103 | 120 | 370 |
| ɤ-GTP (IU/L) |  | 396 | 16 | 73 | 9 | 35 |
| ChE (IU/L) |  | 331 | 173 | 57 | 168 | 470 |
| HbA1c (%) |  | 7.2 | 5.6 | 6.5 | 4.7 | 6.2 |
| Total Cholesterol (mg/dL) |  | 218 | 86 | 177 | 120 | 220 |
| Triglyceride (mg/dL) |  | 161 | 92 | 177 | 30 | 150 |
| High density lipoprotein (mg/dL) |  | 87 | 30 | 36 | 40 | 71 |
| Low density lipoprotein (mg/dL) |  | 114 | 42 | 84 | 65 | 139 |
| Hyaluronic acid (ng/mL) |  | 265 | 110 | - |  | 50 |
| Type IV collagen 7S (ng/mL) |  | 7.9 | 10.9 | - |  | 4.4 |
| M2BPGi (AU/mL) |  | 2.51 | 4.76 | 6.1 |  | 0.84 |
| Complete blood count |  |  |  |  | Lower limit | Upper limit |
| Red blood cell count (/µL) |  | 466×10^4 | 457×10^4 | 445×10^4 | 376 | 500 |
| Hemoglobin (g/dL) |  | 14.3 | 12.6 | 13.2 | 12 | 16 |
| Hematocrit (%) |  | 41.8 | 39.9 | 41.5 | 35.5 | 45 |
| Mean corpuscular volume (fL) |  | 89.7 | 87.3 | 93.3 | 80 | 100 |
| White blood cell count (/µL) |  | 4580 | 4520 | 4300 | 4000 | 9000 |
| Platelet (/µL) |  | 13.0×10^4 | 7.5×10^4 | 7.7×10^4 | 15 | 35 |
| Prothrombine time (%) |  | >100 | 74 | 117.6 | 80 | 130 |
| Prothrombin Time-International Normalized Ratio (%) | | 0.96 | 1.19 | 0.92 |  |  |
| Tumor marker |  |  |  |  |  |  |
| α-fetoprotein (ng/mL) |  | 4.9 | 1.3 | - |  | 10 |
| PIVKA-II (mAU/mL) |  | 23 | 177 | - |  | 40 |
| Immunological test |  |  |  |  | Lower limit | Upper limit |
| IgG (mg/dL) |  | 1,598 | 1,535 | 3,068 | 870 | 1700 |
| IgA (mg/dL) |  | 316 | 203 | 330 | 110 | 410 |
| IgM (mg/dL) |  | 613 | 203 | 189 | 35 | 220 |
| CH50 (IU/mL) |  | 35 | 44 | 33.5 | 30 | 45 |
| C3 (mg/dL) |  | 112 | 77 | 119 | 65 | 135 |
| C4 (mg/dL) |  | 23 | 13.1 | 15 | 13 | 35 |
| Endocrine function test |  |  |  |  | Lower limit | Upper limit |
| Thyroid stimulating hormone (IU/mL) |  | <0.01 | 4.5 | 1.06 | 0.35 | 4.94 |
| Thyroxine (ng/dL) |  | 1.84 | 0.95 | 0.55 | 0.7 | 1.48 |
| TgAb (IU/mL) |  | 11 | 11 | - |  | <28 |
| Anti-thyroglobulin antibody (IU/mL) |  | <9.0 | <9.0 | <9.0 |  | <16 |
| Anti glutamic acid decarboxylase antibody (IU/mL) |  | ＜5.0 | ＜5.0 | ＜5.0 |  | <5 |
| Viral hepatitis |  |  |  |  |  |  |
| HBs Ag |  | Negative | Negative | Negative |  |  |
| HCV antibody |  | Negative | Negative | Negative |  |  |
| Autoantibodies |  |  |  |  |  | Normal |
| Anti-nuclear antibody | Antibody titer | 1,280 | 640 | 5,120 |  | <40 |
|  | Homogeneous | <40 | <40 | <40 |  | <40 |
|  | Speckled | 40 | <40 | 5,120 |  | <40 |
|  | Nucleolar | <40 | <40 | <40 |  | <40 |
|  | Discrete-Speckled | 1,280 | 640 | <40 |  | <40 |
|  | Peripheral | <40 | <40 | <40 |  | <40 |
|  | nuclear envelope | <40 | <40 | <40 |  | <40 |
|  | PCNA | <40 | <40 | <40 |  | <40 |
|  | PCNA-like | <40 | <40 | <40 |  | <40 |
|  | Granular | <40 | <40 | <40 |  | <40 |
|  | Cytoplasmic | Positive | <40 | 640 |  | <40 |
| Anti-centromere antibody |  | 1,280 | 258 (IU/mL) | >500 (IU/mL) |  | <40 |
| Anti-mitochondrial antibody (AMA), IIF titer |  | 160 | <20 | <20 |  | <20 |
| AMA-M2 (index) |  | 59.9 | 45.5 | 30.2 |  | <7.0 |
| Anti-cardiolipin beta-2 glycoprotein I complex antibodies (U/ml) |  | <1.3 | <1.3 | <1.3 |  | <3.5 |
| IgG anti-double-stranded DNA antibody (IU/mL) |  | <10 | - | 4 |  | <12 |
| Anti-ribonucleoprotein antibody |  | Negative  (Ouchterlony method; OM) | - | >550 IU/mL  (EIA) |  | Negative (OM)　 /<10.0  (EIA) |
| anti-SS-A/Ro antibody |  | Negative (OM) | Negative (OM) | >1,200 IU/mL  (EIA) |  | Negative (OM)　 / <10.0  (EIA) |
| SS-B/La antibody |  | Negative (OM) | Negative (OM) | - |  | Negative (OM) |
| anti-Scl-70 antibody |  | Negative (OM) | Negative (OM) | < 1.0 IU/mL  (EIA) |  | Negative (OM)　 / <10.0  (EIA) |
| Clinical Status |  |  |  |  |  |  |
| UDCA at assessment |  | (+) | (+) | (-) |  | N/A |
| Varices / splenomegaly |  | (+) | (+) | (+) |  | (-) |
| LSM / SSM (kPa) |  | 8.5 / N/A | 9.3 / 108.2 | 8.5-9.0 / >75 |  | N/A |
| HVPG (mmHg) |  | <5 | 17, 13 | 5 (median) |  | <5-6 |
| ALP, alkaline phosphatase; ALT, alanine aminotransferase; AMA, anti-mitochondrial antibodies; ANA, anti-nuclear antibodies; AST, aspartate aminotransferase; ɤGTP, gamma glutamyl transpeptidase; HBsAg, hepatitis B surface antigen; HCV, hepatitis C virus; PT-INR, prothrombin time-international normalized ratio.ACA and AMA results were obtained using different assay platforms and are not directly comparable across cases. | | | | | | |
